# Eosinophilic Granulomatous Polyangiitis Presenting With Finger Swelling as the Main Manifestation: A Case Report and Analysis

**DOI:** 10.1002/ccr3.72722

**Published:** 2026-05-17

**Authors:** Lingfang Zhou

**Affiliations:** ^1^ Taizhou Hospital of Zhejiang Province Affiliated to Wenzhou Medical University Taizhou China

**Keywords:** case report, Churg‐Strauss syndrome, elderly patient, eosinophilic granulomatous polyangiitis, finger swelling

## Abstract

EGPA should be considered in elderly patients with asthma presenting with unexplained finger swelling and eosinophilia. Early recognition prevents delayed diagnosis.

## 1 Introduction

Eosinophilic granulomatosis with polyangiitis (EGPA), previously known as Churg‐Strauss syndrome, is a rare systemic vasculitis characterized by asthma, eosinophilia, and vasculitis affecting multiple organ systems. The disease primarily manifests with respiratory symptoms, skin lesions, and peripheral neuropathy, often leading to significant morbidity if not diagnosed and treated promptly. The diagnosis of EGPA is based on clinical criteria, including the presence of asthma, eosinophilia, and evidence of vasculitis, often supported by imaging studies and tissue biopsies that reveal eosinophilic infiltration and necrotizing vasculitis [1]. Current research highlights the complexity of EGPA, emphasizing the need for a high index of suspicion in patients presenting with respiratory symptoms and eosinophilia, particularly in those with a history of asthma.

In this case report, we present a 77‐year‐old male with a significant history of bronchial asthma who developed acute, migratory swelling and pain in both hands, ultimately leading to a diagnosis of EGPA. This case is particularly noteworthy due to the rarity of EGPA in the elderly population and the atypical presentation of symptoms, which may serve as a differential diagnosis in patients presenting with unexplained joint pain and eosinophilia. The findings underscore the importance of considering EGPA in the differential diagnosis of patients with a history of asthma and new‐onset inflammatory symptoms, as early recognition and treatment can significantly improve outcomes.

## 2 Case Presentation

### 2.1 Patient Information

The patient was a man in his 70s with a 7‐year history of bronchial asthma, previously untreated. He was admitted to the geriatric medicine ward due to wandering bilateral hand swelling and pain for 10 days, worsening for 1 day.

### 2.2 Clinical Findings

Ten days prior to admission, the patient experienced sudden swelling and pain in the left middle finger without any apparent cause, with mild limitation of flexion, no pruritus, no fever, and no morning stiffness. This episode lasted for several hours and resolved spontaneously. Thereafter, the patient experienced recurrent, migratory, mild, and self‐resolving swelling and pain in both hands. One day prior to admission, he noted a recurrence of swelling and pain in the right hand, which worsened and was accompanied by skin redness, predominantly affecting the thenar muscles, with difficulty making a fist.

Physical examination on admission:

- Temperature: 37.2°C.
- Pulse: 89 beats/min.
- Respiratory rate: 18 breaths/min.
- Blood pressure: 158/98 mmHg.
- Consciousness: Alert.
- Respiration: Normal.
- Superficial lymph nodes: Not enlarged.
- Lung auscultation: No wheezing or crackles detected.
- Right hand: Swelling with tenderness and localized erythema; left hand: unremarkable; no tenderness in limb joints; scattered purpura noted on the posterior surface of both calves.

### 2.3 Diagnostic Assessment

Four hours post‐admission, right hand swelling continued to worsen, with difficulty in making a fist and finger flexion, and the skin began to exhibit a purplish hue. Approximately 6 h later, the left thumb and thenar muscles were also affected, though to a lesser extent than the right side.

Laboratory results on admission:

- White blood cells: 14.5 × 10^9^/L.
- Absolute eosinophil count: 6.74 × 10^9^/L.
- Eosinophil percentage: 46.5%.
- High‐sensitivity C‐reactive protein: 61.2 mg/dL.

**FIGURE 1:**
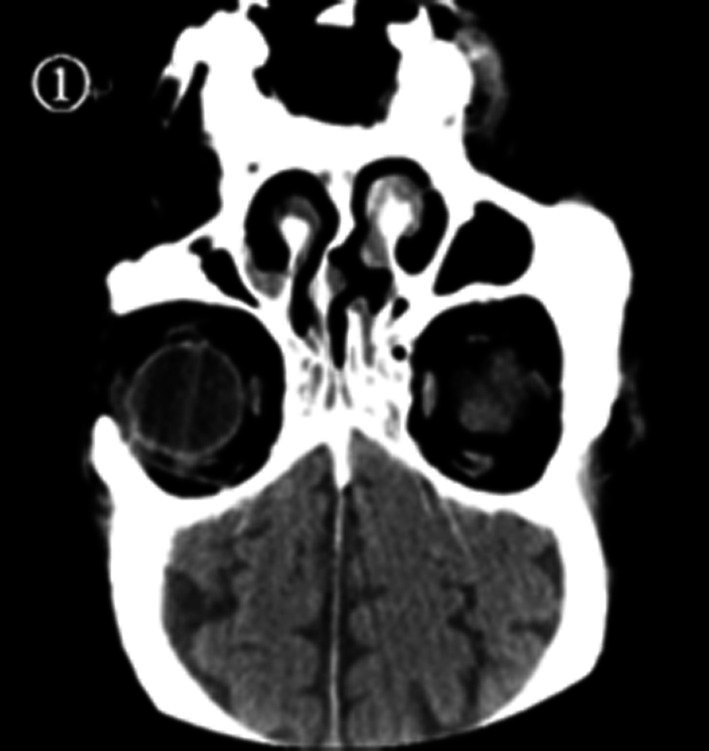
Bilateral paranasal sinus inflammation with slight rightward deviation of the nasal septum.

**FIGURE 2:**
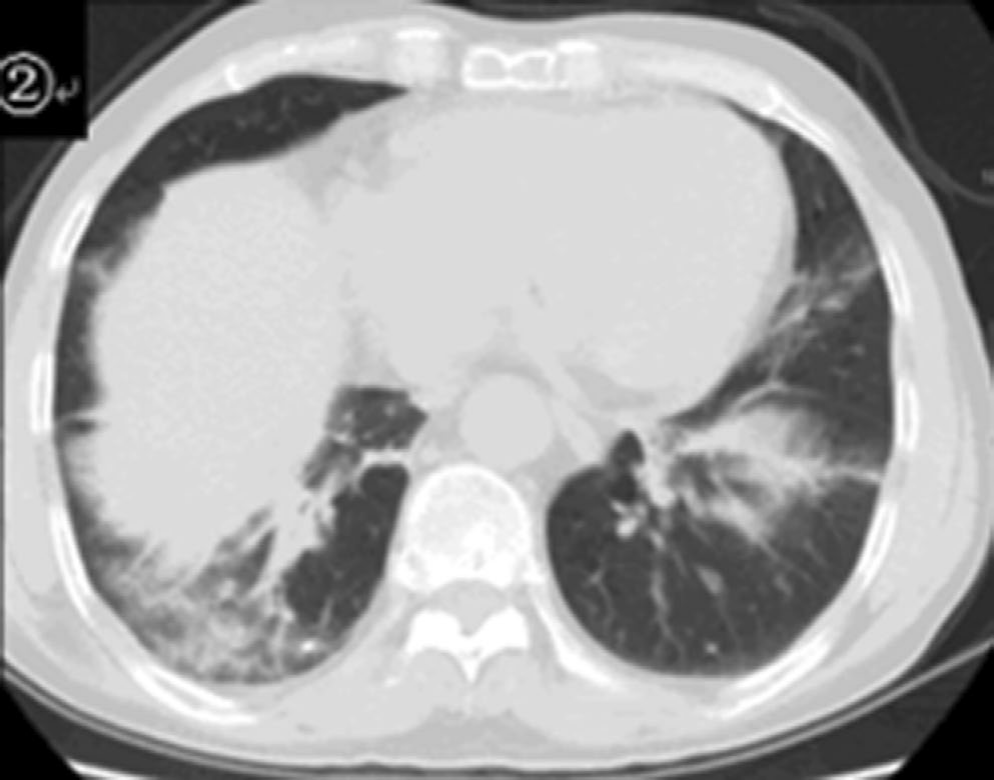
Pneumonic infiltrates in the lingular segment of the left upper lobe and bilateral lower lobes.

Given the patient's history of asthma and significant eosinophilia, clinical suspicion for EGPA was raised. An emergency CT scan of the paranasal sinuses (coronal view) and chest revealed inflammation in both maxillary and ethmoid sinuses, slight rightward deviation of the nasal septum, and lung infiltrates with ground‐glass opacities mixed with consolidation in the left lung upper lobe and both lung lower lobes (Figures 1 and 2).

### 2.4 Differential Diagnosis

Alternative causes of eosinophilia with hand swelling were considered and excluded:

**Table:** 

Differential diagnosis	Exclusion criteria
Hypereosinophilic syndrome (HES)	No specific organ involvement; no vasculitis on histopathology
Parasitic infection	No fever; stool ova/parasite examination negative
Rheumatoid arthritis	Rheumatoid factor and anti‐CCP antibodies negative
Cellulitis	No fever; migratory pattern atypical; rapid response to steroids

### 2.5 Therapeutic Intervention

Due to the rapid progression of the patient's condition, intravenous methylprednisolone (80 mg) was administered daily for 3 days, followed by oral methylprednisolone (48 mg) every morning (1 mg/kg/day), along with omeprazole for gastric protection.

### 2.6 Follow‐Up and Outcomes

Hospital Day 2:

The patient reported reduced pain in the right hand, with complete resolution of swelling in the left hand. Follow‐up laboratory tests showed:

- White blood cells: 10.0 × 10^9^/L.
- Absolute eosinophil count: 0.71 × 10^9^/L.
- Eosinophil percentage: 7.1%.
- Erythrocyte sedimentation rate: 34 mm/h.
- Total IgE concentration: > 2500 IU/mL.
- High‐sensitivity C‐reactive protein: 72.9 mg/dL.
- Liver, renal, thyroid function tests, tumor markers, antinuclear antibodies, ANCA, G test, GM test, and Aspergillus IgG antibodies: Negative.
- Electromyography: Peripheral neuropathy in both lower limbs.
- Pulmonary function tests: Post‐bronchodilator FEV1 at 81% of predicted value, FEV1/FVC ratio 65.38%, mild obstructive ventilatory defect, slight reduction in diffusing capacity, negative bronchodilator test.

**FIGURE 3:**
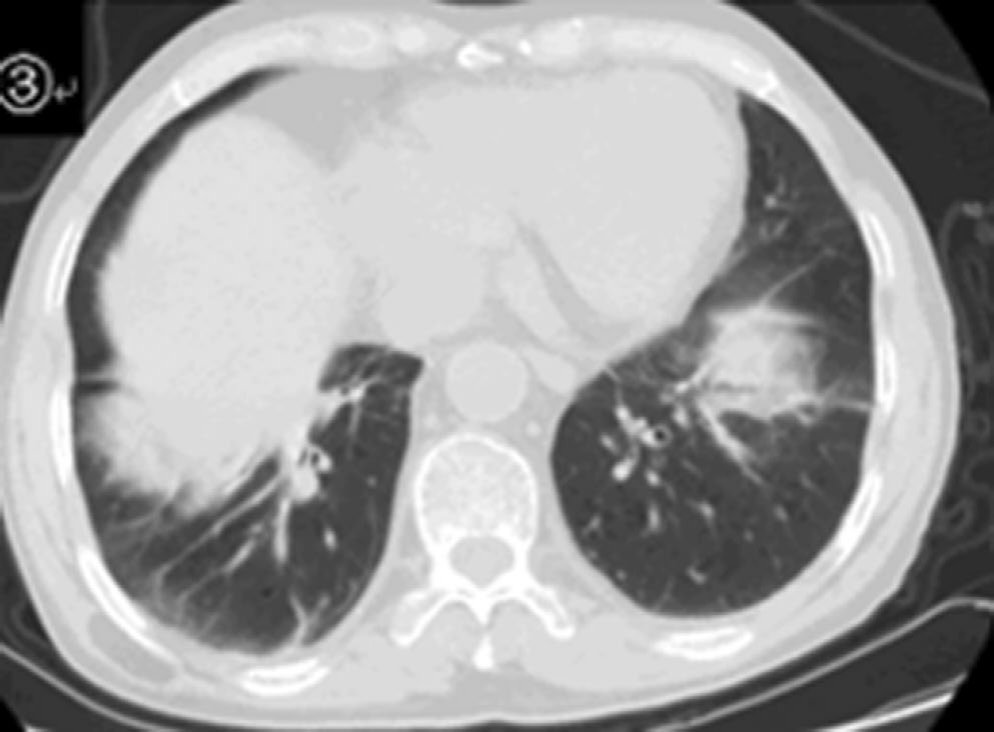
Improvement in the inflammation of the left lung upper lobe and both lung lower lobes.

Hospital Day 3:

Follow‐up chest CT scan showed improvement in the inflammation of the left lung upper lobe and both lung lower lobes (Figure 3).

**FIGURE 4:**
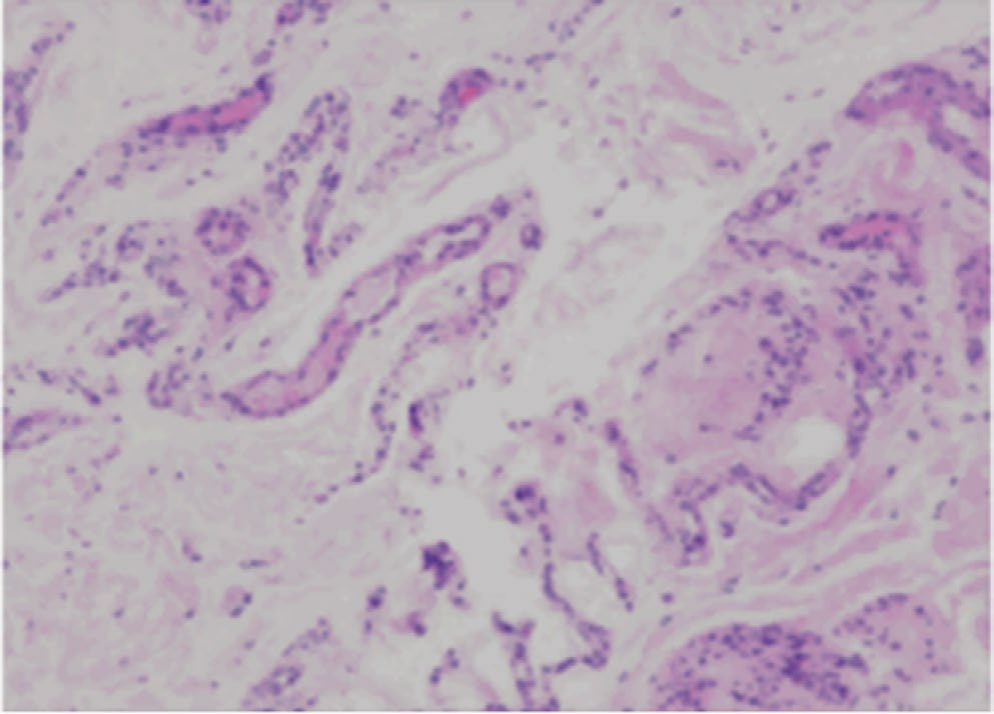
(Left lower leg) A small amount of fibrovascular tissue and skeletal muscle tissue with focal eosinophilic infiltration (HE staining ×200).

Hospital Day 4:

A muscle biopsy of the left calf revealed minimal fibrous vascular tissue and striated muscle tissue with focal eosinophilic infiltration (Figure 4).

**FIGURE 5:**
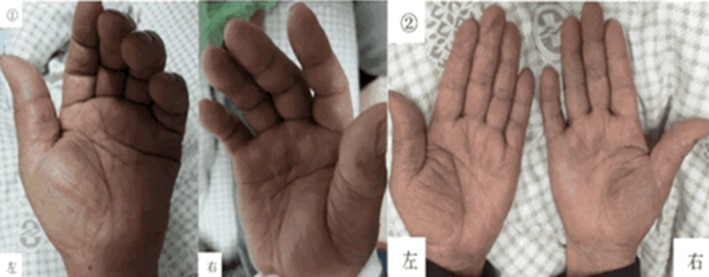
The condition of both hands on Days 1 and 5.

Hospital Day 5:

Bilateral hand pain and lower limb rash had largely resolved (Figure 5).

Hospital Day 7:

Repeat laboratory tests indicated:

- White blood cells: 6.3 × 10^9^/L.
- Absolute eosinophil count: 0.37 × 10^9^/L.
- Eosinophil percentage: 5.9%.
- Total IgE concentration: 1527 IU/mL.

The patient's clinical symptoms improved significantly. He was discharged on maintenance oral corticosteroids and followed up regularly in the outpatient clinic.

## 3 Discussion

A structured literature search conducted in PubMed and CNKI databases using the keywords “EGPA” or “Churg‐Strauss” or “Eosinophilic granulomatous polyangiitis” and “finger swelling” found no relevant articles.

The presented case of a man in his 70s with a history of bronchial asthma who developed migratory hand swelling and pain, ultimately diagnosed with EGPA, is of significant clinical interest. This case exemplifies the intricate relationship between underlying respiratory conditions and the onset of systemic vasculitides, highlighting the need for comprehensive evaluations in patients with atypical presentations.

Current literature on EGPA emphasizes a spectrum of clinical manifestations often associated with eosinophilia, asthma, and systemic vasculitis. The patient's initial symptoms of finger swelling without significant systemic involvement align with reported presentations of EGPA. The rapid progression of symptoms in this case underscores the aggressive nature of EGPA and the urgency in diagnosis and management, particularly in patients with prior respiratory conditions [2].

The core mechanism of EGPA presenting with hand swelling may include small/medium vessel involvement in the hand and eosinophil activation leading to local inflammation, increased permeability, and impaired drainage.

In terms of diagnostic processes, the combination of clinical symptoms, elevated eosinophil counts, and imaging studies played a critical role in confirming the diagnosis. The patient's significant eosinophilia, alongside the characteristic imaging findings of pulmonary involvement, reflects the typical diagnostic criteria for EGPA [3, 4]. Furthermore, the rapid response to corticosteroid therapy highlights the effectiveness of glucocorticoids in controlling inflammation and preventing further complications in EGPA patients [5].

The treatment strategy employed in this case, involving high‐dose intravenous methylprednisolone followed by oral administration, is consistent with current recommendations for managing EGPA. The observed improvements in symptoms and laboratory parameters post‐treatment further support the efficacy of corticosteroids in this context [5]. Monitoring for potential side effects and ensuring patient safety during corticosteroid therapy remains paramount, especially in older adults with comorbid conditions.

In this case, following corticosteroid treatment, the patient's IgE levels showed a continuous downward trend, paralleling improvements in clinical symptoms (such as asthma, rash, and peripheral blood eosinophil counts), suggesting effective control of disease activity. Previous studies have also shown that in patients with EGPA, a decrease in IgE during treatment is associated with a reduced risk of relapse and a higher success rate of glucocorticoid tapering.

## 4 Conclusion

This case contributes to the growing body of evidence linking respiratory conditions, such as asthma, with systemic eosinophilic diseases like EGPA. It highlights the importance of vigilant clinical assessment, timely intervention, and continued research to enhance our understanding of these complex interactions and improve patient care in this challenging clinical landscape. Clinicians should consider EGPA in elderly patients with asthma and unexplained hand swelling accompanied by eosinophilia.

## Author Contributions

**Lingfang Zhou:** data curation, formal analysis, writing – original draft, writing – review and editing.

## Funding

The author has nothing to report.

## Data Availability

Data sharing is not applicable to this article as no new data were created or analyzed in this study.
